# Combined Total Knee Arthroplasty and Medial Patellofemoral Ligament Reconstruction for Chronic Patellar Dislocation and Severe Osteoarthritis

**DOI:** 10.1016/j.artd.2020.11.024

**Published:** 2020-12-26

**Authors:** Ignacio Garcia-Mansilla, Kristofer J. Jones, Adam A. Sassoon

**Affiliations:** a Knee Division, Hospital Italiano de Buenos Aires, Buenos Aires, Argentina; b Department of Orthopaedic Surgery, Division of Sports Medicine and Shoulder Surgery, David Geffen School of Medicine at UCLA, Los Angeles California, Los Angeles, CA, USA; c Department of Orthopaedic Surgery, David Geffen School of Medicine at UCLA, Los Angeles California, Los Angeles, CA, USA

**Keywords:** Patellar dislocation, TKA, Medial patellofemoral ligament reconstruction

## Abstract

Chronic patellar dislocation in the setting of severe knee osteoarthritis is a rare clinical problem. Surgical management often consists of total knee arthroplasty combined with realignment of the extensor mechanism. Several techniques have been described to anatomically restore the extensor apparatus, and literature regarding this topic consists mainly of case reports. We describe a technique using combined medial patellofemoral ligament reconstruction using allograft tissue and total knee arthroplasty with patellar resurfacing for the treatment of chronic patellar dislocation and severe osteoarthritis.

## Introduction

Chronic patellar dislocation is recognized as a rare entity that can be due to congenital conditions but can also be acquired secondary to trauma. In the setting of severe osteoarthritis (OA), a fixed patellar dislocation represents a challenge to the orthopedic surgeon, and there is no consensus regarding treatment of these patients [[1], [2], [3]]. Realignment of the extensor mechanism is mandatory to restore the biomechanics of the knee and recover active extension. Moreover, suboptimal patellofemoral alignment after total knee arthroplasty (TKA) is associated with poor clinical outcomes [4].

Functional patellar tracking in most primary TKA is achieved with optimal positioning of the femoral and tibial components [5]. A number of surgical techniques may be applied to enhance patellar tracking during total knee replacement, including correction of coronal alignment [6], proper patellar thickness (avoidance of “overstuffing”) [7], external rotation of the femoral and tibial components [8,9], lateralization of the femoral component and medialization of the patellar component, or lateral soft tissue release [10]. However, in the setting of chronic patella dislocations, the aforementioned techniques may not be sufficient, and persistent maltracking is observed. In these cases, proximal, distal, or combined realignment of the extensor mechanism is necessary to achieve a satisfactory clinical outcome.

Various approaches have been proposed to address fixed patellar dislocations in the setting of knee OA, and literature regarding this topic consists mainly of case reports that describe varying realignment techniques combined with TKA [1,3,[11], [12], [13], [14], [15], [16]]. This technique article describes the utilization of a soft tissue allograft to reconstruct the medial patellofemoral ligament (MPFL) along with an extensive lateral release to achieve soft tissue balancing for a patient with a fixed patellar dislocation and knee OA.

## Surgical technique

### Patient background

**Figure 1:**
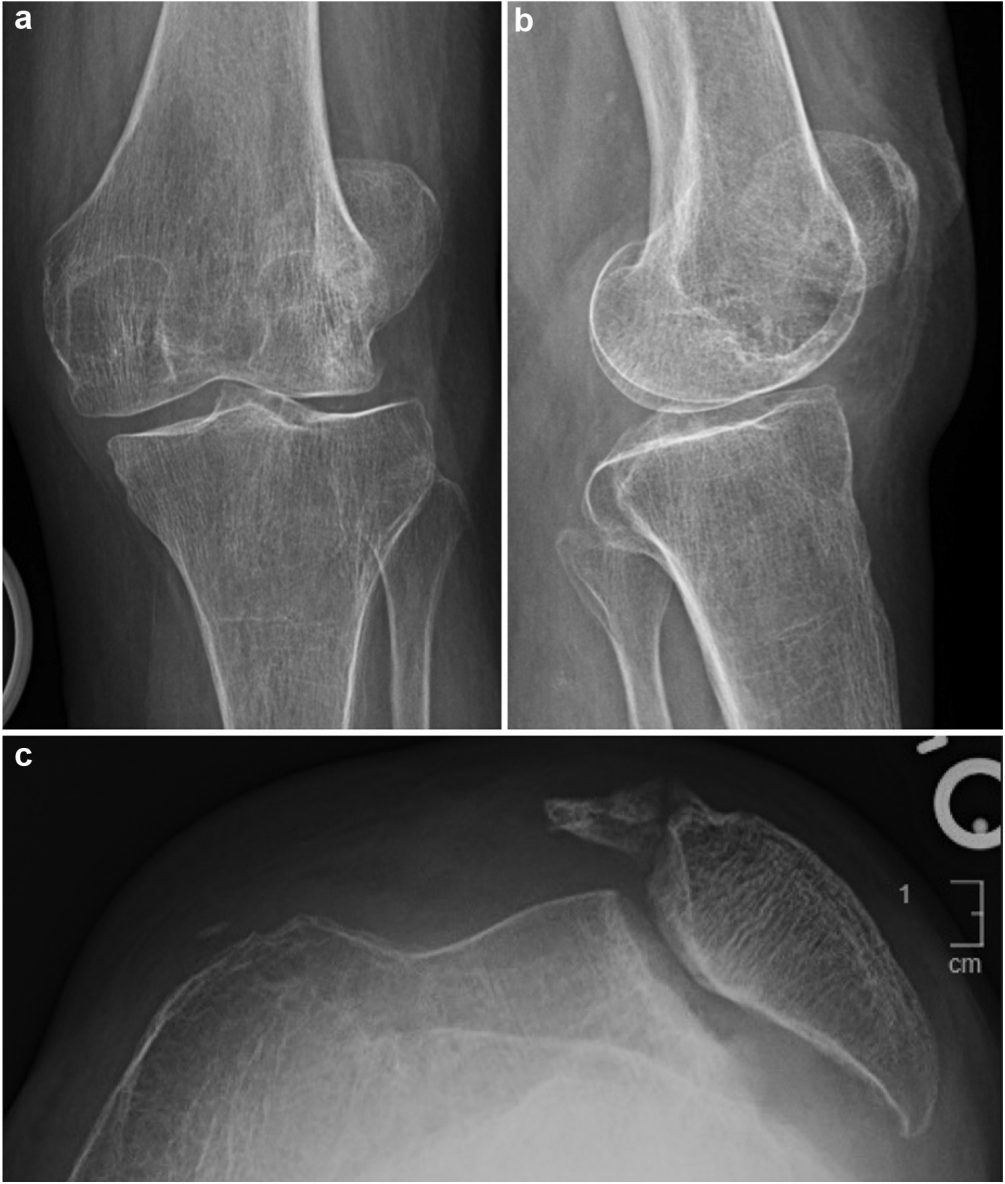
Preoperative X-rays of the left knee. Anteroposterior (a) and lateral (b) views demonstrate osteoarthritis with lateral dislocation of the patella. Merchant view (c) shows laterally dislocated patella.

The patient is a 70-year-old woman with a longstanding history of left knee pain secondary to a chronic patellar dislocation. She sustained a left knee injury 1 year before surgery and complained of diminished range of motion since that time. Physical examination revealed neutral alignment with a flexion extension arc that measured from 10° to 75°. Radiographs revealed a completely dislocated patella and notable irregularity of the trochlear and patellar contour (Fig. 1c). Both medial and lateral compartments demonstrated joint space narrowing and osteophyte formation (Fig. 1a and b).

### Surgical approach

**Figure 2:**
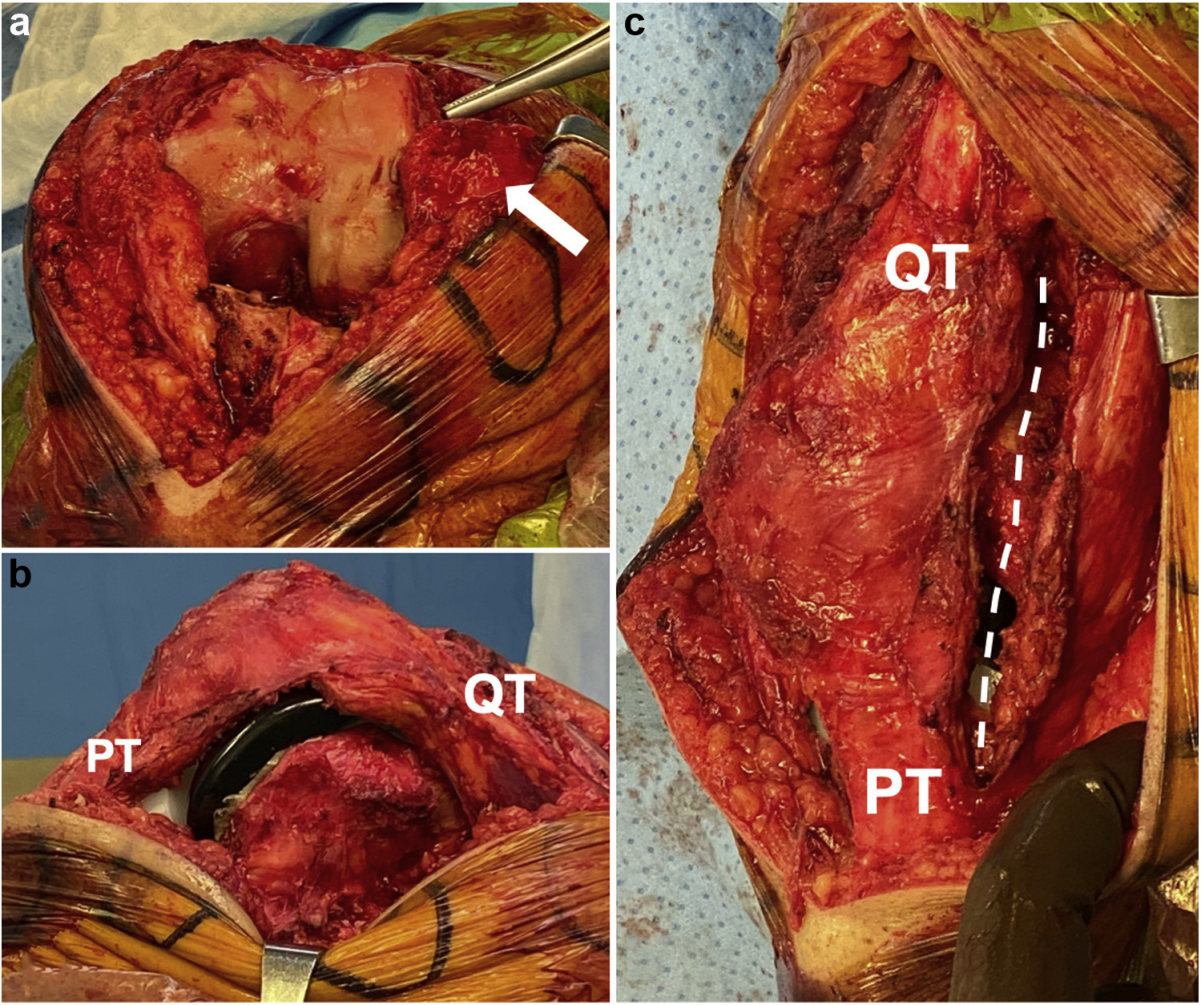
Left knee intraoperative images. Anterior view (a) shows an extended medial parapatellar arthrotomy with medial release of the soft tissues along the proximal tibia. Patella is dislocated laterally (white arrow). Lateral (b) and anterior (c) view after an extensive lateral release (white dotted line) performed to mobilize the patella. PT = patellar tendon; QT = quadriceps tendon.

The patient was positioned supine on the operating table, and an anterior midline incision was performed. The extensor mechanism was then identified, and it was noted that the white-red junction of the quadriceps tendon and the vastus medialis was extremely lateral because of the chronic patellar dislocation. An extended medial parapatellar arthrotomy was performed followed by a medial release of the soft tissues along the proximal tibia (Fig. 2a). To mobilize the patella, an extensive lateral release was performed (Fig. 2b and c).

### Total knee arthroplasty

A TKA is performed in a standard fashion implementing a series of technical pearls to improve patellar tracking. The femoral component was positioned in 5° of external rotation, and the tibial component was also externally rotated relative to the patellar tendon. In this case, the patella was resurfaced with a 7.5-mm polyethylene insert to avoid overstuffing the patellofemoral compartment. The patellar bone and implant composite should be 1 to 2 mm thinner than the native patella to improve flexion and patellar tracking. In addition, the patellar component was lateralized to position the apex of the patella within the trochlear groove more effectively. The patellar component was also moved slightly inferior to avoid convergence with the suture anchors that would eventually be inserted for the MPFL reconstruction. A cruciate-retaining Legion, Oxinium, femoral component and all-polyethylene tibial component (Smith and Nephew, Memphis, TN) were used. Patellar tracking was reassessed through range of motion and using the so-called no-thumb technique [17] after tourniquet release. A tendency for patellar dislocation was noted with passive knee flexion; therefore, MPFL reconstruction was performed.

### MPFL reconstruction

**Figure 3:**
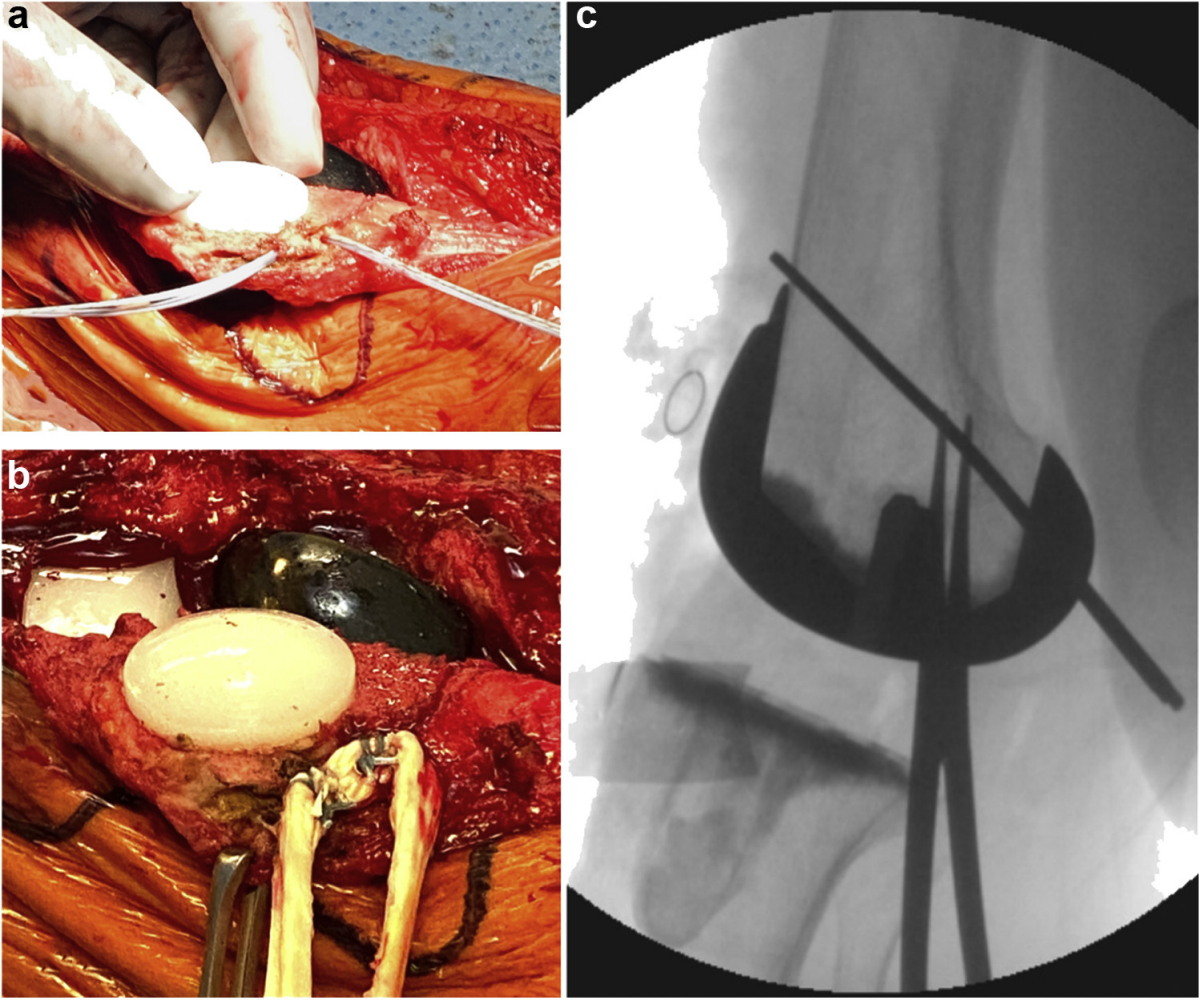
Left knee intraoperative images. Lateral view (a), the patella is everted and two 3.0-mm anchors are inserted at the junction of the superior one-third and inferior two-third of the patella. Tibialis anterior allograft folded onto itself and secured to the medial border of the patella with locked sutures (b). Fluoroscopic examination revealed appropriate placement for the femoral tunnel at the location of Schoettle's point (c).

**Figure 4:**
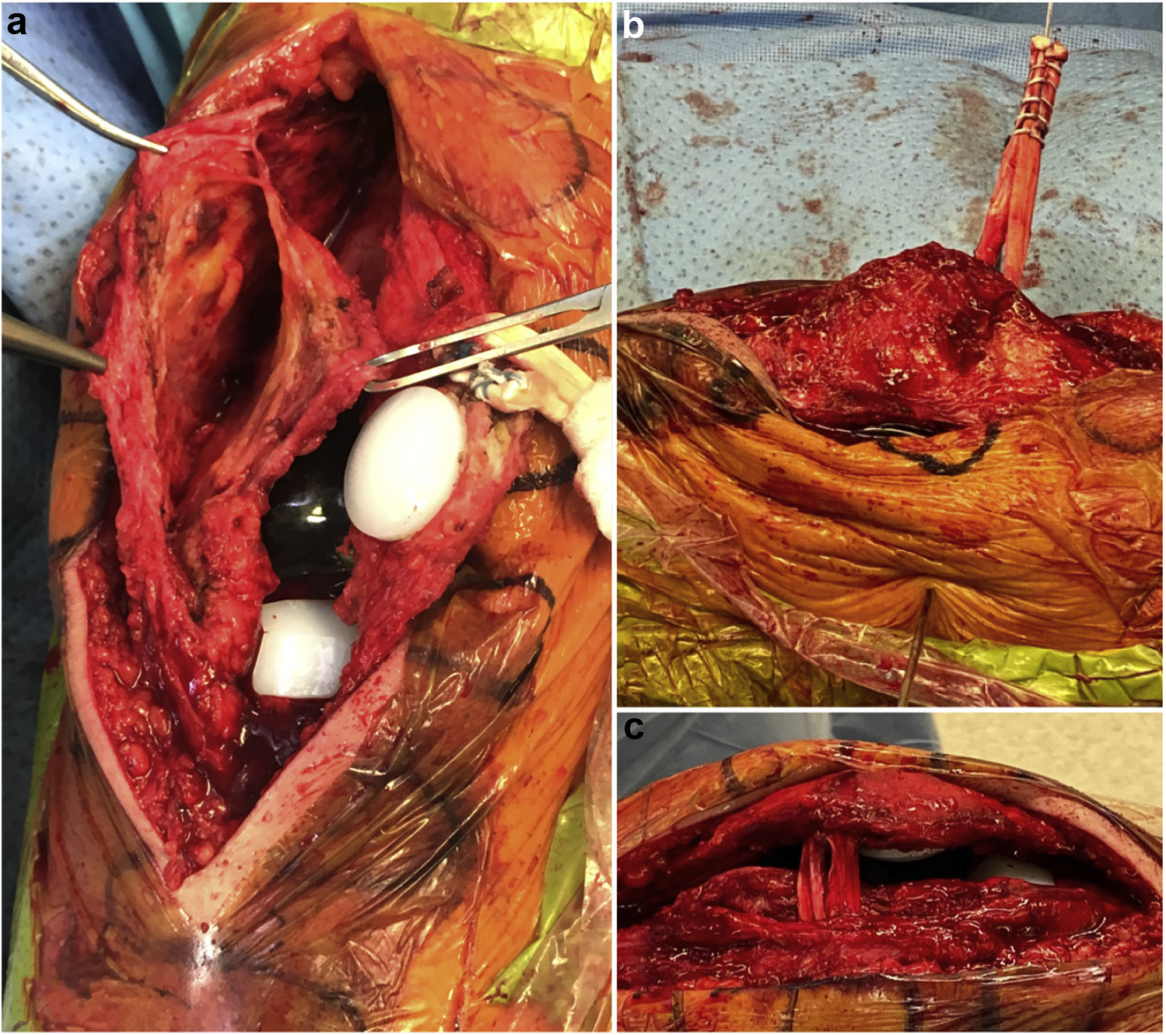
Left knee intraoperative images. Anterior view (a) showing dissection of layers 2 and 3 of the medial side of the knee. Lateral view (b) shows the pin drilled for the femoral tunnel. Medial view (c) showing final graft positioning.

**Figure 5:**
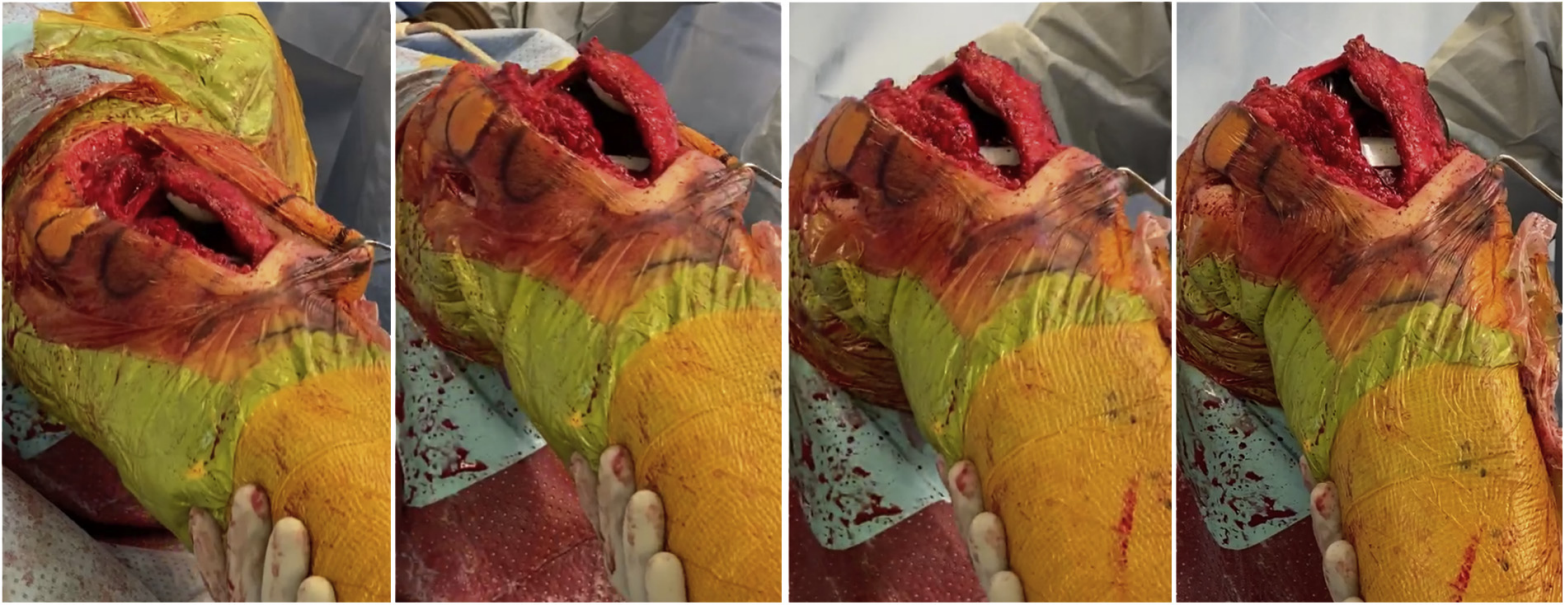
Left knee intraoperative images. Appropriate patellar tracking is assessed through complete range of motion (0°, 45°, 90°, 110°).

The medial border of the patella was exposed with electrocautery to identify the native footprint of the MPFL. Two 3.0-mm double-loaded biosuture tack anchors (Arthrex, Naples, FL) were inserted 1 centimeter apart at the junction of the superior one-third and inferior two-third of the patella. The drill holes were directed away from the patellar component pegs to avoid iatrogenic damage which could compromise component fixation (Fig. 3a). A tibialis anterior allograft was subsequently looped/folded onto itself, and the longitudinal aspect of the tendon was secured to the medial border of the patella with locked sutures. These sutures were then tied down, and the graft was tested to ensure secure fixation (Fig. 3b). A separate 3-cm incision was made along the medial knee and carried down to the level of the deep fascia. The sulcus between the medial epicondyle and adductor tubercle was identified. An 18-g needle was inserted into the bone in the area of Schoettle's point [18] and fluoroscopic examination revealed appropriate placement for the femoral tunnel. A beath pin was inserted and drilled in a medial to lateral direction, aimed anteriorly and proximally (Figure 3, Figure 4b). A curved clamp was used to pass a shuttle suture between layers 2 and 3 of the knee (Fig. 4a), and the graft is passed so the tails exit the posterior incision. A single #2 FiberLoop suture was used to tag each graft end (Fig. 4b), and the graft was sized. In our case, the graft measured 8 mm, so an 8-mm low-profile reamer was used to create the femoral tunnel. The depth of the tunnel should be approximately 25 to 30 mm. The Fiberloop sutures are inserted into the end of the beath pin and shuttled to the lateral side of the femur. The leg is flexed to 30°, and the graft is appropriately tensioned so that the patella is centered in the trochlear groove. A 7 × 23-mm biocomposite (Arthrex) screw is used for femoral fixation. The knee must be able to achieve terminal extension and appropriate flexion based on preoperative examination. In addition, the patella should track normally within the femoral trochlea during passive knee flexion/extension (Fig. 5). Finally, one quadrant of translation should be easily obtained with the knee in full extension to ensure that the graft is not overly tensioned.

Given the increased risk of postoperative infection secondary to longer surgical time, a history of previous surgery, and the use of an allograft, 10 cc of Stimulan beads (Biocomposites Inc., Willmington, NC) were mixed on the back table with 1 g of powdered vancomycin and 1.2 g of powdered tobramycin and then placed in the joint space as a means of antibiotic prophylaxis.

### Closure

**Figure 6:**
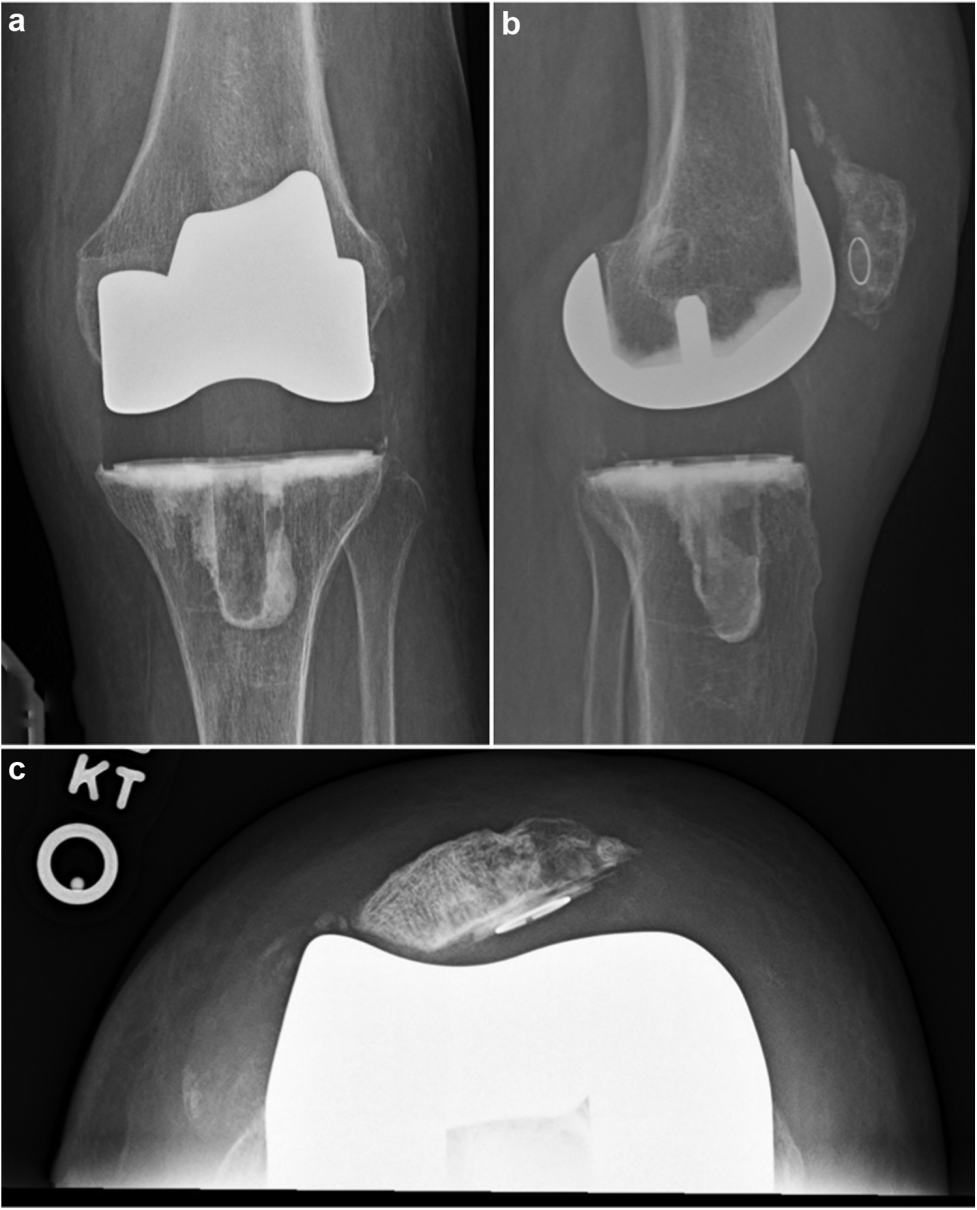
Six-months follow-up X-rays. Anteroposterior (a), lateral (b), and merchant view (c). She has active range of motion from 0° to 115° of flexion. She does have notable lateral patellar tilt in her trochlear groove, but the patella remains centered over the trochlea despite the excessive lateral tilt. No evidence of subluxation or frank dislocation.

The medial retinaculum and the medial parapatellar arthrotomy were repaired in a “pants-over-vest” fashion with #1 Vicryl suture and oversewn with #2 Quill-type suture. A 10 Hemovac drain was placed through the lateral release, exiting laterally in the thigh. Subcutaneous tissues were closed using interrupted 2-0 Vicryl suture. The subcuticular layer was closed with interrupted 2-0 Monocryl, and the skin was closed with 2-0 nylon in a vertical mattress fashion. Immediate postoperative radiographs and those taken at 6 months follow-up (Fig. 6) were obtained.

### Postoperative protocol

After surgery, the patient was placed in a hinged knee brace and allowed to bear weight as tolerated with the brace locked in extension. The patient was allowed to perform range of motion exercises, while in the brace, beginning with 0-30° at 2 weeks postoperatively with weekly advancement of 10°. Weight bearing in an unlocked brace was allowed at 6 weeks and out of the brace at 12 weeks.

## Discussion

Chronic patellar dislocation and concurrent OA is unusual and imparts considerable functional disability. Whether congenital or acquired in etiology, there is no clear treatment protocol for the management of these patients. This case report serves to demonstrate the surgical technique of combined MPFL reconstruction and TKA for the treatment of an acquired chronic patellar dislocation and severe OA.

Several cases of knee OA and chronic patellar dislocation treated with TKA, with or without realignment of the extensor mechanism, have been reported [2,11,12,15]. Some authors published cases successfully treated with TKA, lateral retinaculum release, vastus medialis advancement, and patellar resurfacing [11,15]. Others performed a proximal realignment with the use of a V-W quadriceplasty (quadriceps turndown and medial advancement) [12].

Proximal realignment may be sufficient to treat some of these cases, as shown by Bullek et al., who reported 3 cases treated with a modified proximal patellar realignment [13]. Satisfactory results were obtained with an average follow-up of 40 months. Each patient achieved a well-centralized patella in the trochlear groove, and postoperative outcomes revealed a mean Hospital for Special Surgery Knee-Rating Scale score of 83. There was one complication though, a full-thickness skin necrosis requiring flap coverage. Constrained prostheses were used in all cases because of severe valgus deformity. In a similar way, Kumagai et al. published a case treated with lateral release and medial patellar retinacular plication combined with TKA [14]. One potential disadvantage regarding the use of a medial parapatellar approach combined with a lateral release is the potential devascularization of the patella, as the medial parapatellar approach splits and detaches a portion of the quadriceps from the patella [19]. To overcome this issue, some authors have described the utilization of a subvastus approach in these cases [20,21].

Although tibial tubercle osteotomy and medialization is a well-established treatment for recurrent patellar dislocation, this is a challenging technique to use in conjunction with TKA, and related complications range from 4% to 20%. Tibial plateau fractures, tibial tubercle fractures, skin necrosis, and nonunion are the most common complications associated with this procedure [22,23]. Figueroa et al. published a case of chronic patellar dislocation associated with tricompartmental arthritis treated with TKA, tibial tubercle osteotomy, and vastus medialis advancement [1]. Excellent mid-term results were reported without complications. On the contrary, Yamanaka et al. published a case report of a patient with bilateral congenital dislocated patella treated with a different method in each knee [2]. The first knee was treated with tibial tubercle osteotomy, but a tibia fracture occurred just below the site of the osteotomy. Therefore, the second knee was treated without distal realignment and reduction of the patella.

As we have shown, reinforcement with the MPFL is a useful alternative. Two case reports have described this approach in cases of chronic patellar dislocation and OA. Matsushita et al. [16] reported a case of a 44-year-old woman using semitendinosus tendon autograft and similar graft fixation (2 anchors in the patella and an interference screw in femur). On the other hand, Sato et al. [24] used the artificial Leeds-Keio ligament passed from top to bottom and around the lateral border of the patella. The graft was fixed on the femur using a spiked washer and cancellous screw.

## Summary

In conclusion, chronic patellar dislocation in combination with OA is a rare and complex problem that typically requires a proximal, distal, or combined realignment of the extensor mechanism. In order to achieve successful results, the surgeon should rely on preoperative examination and radiographic imaging to determine the optimal method of realignment, as soft tissue and bony abnormalities should be appropriately addressed to achieve optimal patellofemoral tracking. We believe that combined MPFL reconstruction and TKA is a viable option for the treatment of chronic patellar dislocation and severe OA and avoids potential patient morbidity that may be incurred with distal realignment (osteotomy) procedures.

## Conflict of interests

K. Jones received educational support from Arthrex Inc.; is a consultant for JRF Ortho and Vericel; received research support from Aesculap and Musculoskeletal Transplant Foundation (MTF); and is a board/committee member for American Orthopedic Society for Sports Medicine (AOSSM). A. Sassoon is a consultant for Smith and Nephew, Biocomposites, and Orthalign and is a board/committee member for AAHKS.

For full disclosure statements refer to https://doi.org/10.1016/j.artd.2020.11.024.

## Supplementary data

Conflict of Interest Statement for Sassoon

Conflict of Interest Statement for Garcia-Mansilla

Conflict of Interest Statement for Jones
