# Supplementary material for: Combined Total Knee Arthroplasty and Medial Patellofemoral Ligament Reconstruction for Chronic Patellar Dislocation and Severe Osteoarthritis
Source: Arthroplast Today. 2020 Dec 26;7:47–53. doi: 10.1016/j.artd.2020.11.024 (PMC7818631; doi:10.1016/j.artd.2020.11.024)
Supplement: Conflict of Interest Statement for Jones [file mmc3.docx]

# CONFLICT OF INTEREST STATEMENT

***American Association of Hip and Knee Surgeons***

(Adopted from the American Academy of Orthopaedic Surgeons disclosure statement)

The following form **must be filled out completely and submitted by each author (example, 6 authors, 6 forms).**

**All items require a response. If there is no relevant disclosure for a given item, enter "*None*.”**

Manuscript Title: **Combined Total Knee Arthroplasty and Medial Patellofemoral (MPFL) Reconstruction for Chronic Patellar Dislocation and Severe Osteoarthritis**

1. Royalties from a company or supplier (The following conflicts were disclosed) NONE

2. Speakers bureau/paid presentations for a company or supplier (The following conflicts were disclosed) NONE

3A. Paid employee for a company or supplier (The following conflicts were disclosed) NONE

3B. Paid consultant for a company or supplier (The following conflicts were disclosed)

JRF ORTHO

VERICEL

3C. Unpaid consultants for a company or supplier (The following conflicts were disclosed) NONE

4. Stock or stock options in a company or supplier (The following conflicts were disclosed) NONE

5. Research support from a company or supplier as a Principal Investigator (The following conflicts were disclosed)

AESCULAP

MUSCULOSKELETAL TRANSPLANT FOUNDATION (MTF)

6. Other financial or material support from a company or supplier (The following conflicts were disclosed)

ARTHREX INC (educational support)

7. Royalties, financial or material support from publishers (The following conflicts were disclosed) NONE

8. Medical/Orthopaedic publications editorial/governing board (The following conflicts were disclosed) NONE

9. Board member/committee appointments for a society (The following conflicts were disclosed)

AOSSM

**Each author must sign AND print or type his/her name, date and submit a separate form**

In addition, one BLINDED Conflict of Interest form (no author names used) should be submitted per manuscript with all author disclosures.

KRISTOFER JONES KJ 08/01/2020

Author Name (Print or Type) Author Signature Date
